# The role of external factors on the reactivation of the heritage language of Turkish-German returnees

**DOI:** 10.3389/fpsyg.2023.1156779

**Published:** 2023-12-01

**Authors:** Elena Antonova-Unlu, Fatih Bayram

**Affiliations:** ^1^Department of Foreign Language Education, Hacettepe University, Ankara, Türkiye; ^2^Department of Language and Culture, UiT The Arctic University of Norway, Tromsø, Norway

**Keywords:** heritage language, returnees, external factors, language reactivation, bilingualism, Turkish-German heritage speakers, language performance, heritage language maintenance

## Abstract

**Introduction:**

This study investigates the heritage language performance of Turkish-German returnees upon their reintegration into Turkey and explores the impact of external factors on their proficiency in the (re-)activated heritage language (HL).

**Methods:**

Data collection involved the participation of 28 Turkish heritage speakers and a control group of 28 monolingual speakers. The language proficiency of both groups was assessed through a cloze test and an error correction task with a focus on converbial constructions, evidentiality and direct object case marking in Turkish. A sociolinguistic background questionnaire was used to obtain information about their language experiences. The study focused on understanding the individual and group differences in returnee’s heritage language performance. Additionally, random forest analysis was employed to investigate the relative influence of external factors on individual variability within the returnee group.

**Results and Discussion:**

The analysis of results revealed notable group differences between the returnees and the control group, emphasizing the unique linguistic challenges faced by those who returned to Turkey. Within the returnee group, there was considerable individual variability in heritage language performance. The subsequent exploration of individual variation highlighted the significant role of external factors. Notably, the length of residence in Germany, the age at which participants returned to Turkey, and the frequency of Turkish language use in their migration context emerged as significant predictors of the returnee participants’ proficiency in their (re-)activated HL. Surprisingly, formal contact with the dominant German language did not exert a substantial impact on the returnees’ language proficiency, suggesting the nuanced influence of various external factors on heritage language development.

## Introduction

Returnees represent a unique subset of heritage speakers, typically born in an immigrant setting or spending a significant portion of their early childhood and/or adulthood in that setting before returning to their home or heritage country (Yoshitomi, 1999; Flores, 2020; Flores and Snape, 2021). Much like heritage speaker bilinguals, returnees grow up as bi−/multilingual individuals. They acquire one or more languages as their heritage language, primarily used within their home environment, while they are exposed to the societal language in the broader community, particularly through their schooling. This pattern often results in varying outcomes in their heritage language, typically differing from the established baselines, and the societal language tends to become dominant (for an extensive review, see Montrul, 2016, 2022; Polinsky, 2018). What sets returnees apart is their unique journey of reactivating or relearning their heritage language, leading to a shift in dominance from their former dominant language to their heritage language. This shift occurs as a result of immersion in the home language context upon returning to their home country.

Existing literature on heritage speaker bilinguals consistently underscores the defining characteristic of their linguistic competence in their heritage language: individual variation. Heritage speakers are a diverse group, with their linguistic competence spanning a wide spectrum of differences shaped by their specific contexts and experiences with the languages around them (see, for example, Gathercole and Thomas, 2005; Rodina and Westergaard, 2015; Unsworth, 2015; Montrul, 2016; Correia and Flores, 2017; Antonova-Unlu and Wei, 2018; Kupisch and Rothman, 2018; Armon-Lotem and Meir, 2019; Polinsky and Scontras, 2019, 2020; Bayram et al., 2021; Montrul, 2022). When returnees, who, by nature, are former heritage speakers, settle in their new home country environment, the exposure to the home country language, which is now the dominant societal language, has consequences for their linguistic competencies in both their heritage language and their second language—the societal dominant language in their former country of residence. Similar to the context of typical heritage language bilinguals, the context in which returnees continue to live in their new home country plays a crucial role in determining their linguistic outcomes.

Therefore, the interesting question that arises is whether all returnees end up with the same linguistic outcome in their heritage language. What happens to the HL when HSs return to their country of origin? Will the potential variations in the HL exist after many years spent in an environment where their HL is dominant? If there is still variation, what experiential factors will predict the attainment in the (re-)activated HL? The present study aims to examine these questions, focusing on the (re-)activation of the (re-)activated heritage Turkish of Turkish-German returnees.

## (Re-)activation of returnees’ HL

Returning to the home country entails one significant linguistic consequence, namely the transition of the once-heritage language back to the dominant societal language. This shift naturally offers various advantages, e.g., among others, increased exposure to a broader spectrum of formal and informal language use with an increased number of opportunities for interaction with various interlocutors. Limited research suggests that the reactivation of the heritage language yields positive outcomes. For instance, within the first year of returning for lexicon, and, on average, after approximately 7 years for morphosyntax, the heritage language of returnees can become nearly indistinguishable from that of monolingual speakers in their home country (Daller and Yıldız, 1995; Treffers-Daller et al., 2016).

However, it is also important to recognize that not all aspects of the heritage language may fully reactivate at the same level (Treffers-Daller et al., 2007; Kaya-Soykan et al., 2023). For instance, in the context of heritage Turkish, Treffers-Daller et al. (2007) examined the use of syntactic embeddings by Turkish-German bilinguals residing in Germany, Turkish-German returnees who had been living in Turkey for 8 years, and Turkish monolinguals. This study revealed that Turkish-German bilinguals used fewer and less complex embeddings than the returnee group. While some returnees exhibited similar performance outcomes to the control group, both the heritage speaker group and the returnees, on average, performed relatively worse than the monolingual control group. Similarly, Kaya-Soykan et al. (2023) looked at the use of evidentiality markers in the heritage Turkish of Turkish-German returnees who returned to Turkey as adults and had resided in the country for more than a decade. The findings show differences in the heritage Turkish of the returnees compared to the Turkish control group. Even after many years of residence in their home country, the returnees preserved features typically found in heritage speakers.

To our knowledge, only two studies have explored the factors influencing competence in the reactivated heritage language. Flores and Rato (2016) examined the accent of returnees in their heritage language and found that the age at which they immigrated to the host country, Germany, influenced the variability in their heritage language pronunciation, while the length of residence in the home country post-return did not. In a more recent study, Kubota et al. (2021) investigated the narrative skills of Japanese-English returnees in their heritage Japanese immediately upon return and after 1 year of residence in the home country. Their findings indicated that the age at which the returnees returned and their relative proficiency in the heritage language predicted developments in their heritage language skills within the first year after returning.

## Current study

The present study aims to examine the linguistic attainment of Turkish-German returnees in their (re-)activated heritage Turkish after several years (*M* = 18.96, *SD* = 12.05) of residence in the country of origin and the role of external factors in the attainments of the (re-)activated HL.

The study aims to answer the following research questions:

1. What are, if any, the differences between the (re-)activated HL Turkish and the baseline Turkish?
2. What external factors modulate the attainment in the (re-)activated HL of the returnee participants?

To assess the attainment in the (re-)activated HL of the returnees, we examine their overall use of the Turkish language using a c-test as well as an error correction task, including morphosyntactic structures (converbs, evidentiality, and direct object marking) that have been reported as vulnerable in Turkish (see for a review, Arslan et al., 2015; Antonova-Unlu, 2015; Antonova-Unlu and Wei, 2020; Bayram, 2020) as well as other HLs (Montrul and Polinsky, 2011; Polinsky and Scontras, 2019).

### Converbial constructions

A converb is a non-finite verb form that marks adverbial subordination (Haspelmath, 1995, p. 3) operating as a clause-linking device (Coupe, 2006). From a syntactic point of view, converbs are divided into strict and non-strict (Nedjalkov, 1998). Strict converbs are used with an adverbial function only. Non-strict converbs represent forms derived from participles, verbal nouns, or infinitives, and that is why they are often called participles or gerunds used as an adverb. Turkish converbs are strict and non-finite verb forms that function to express time, manner, purpose and result, cause, condition, degree, place, and concession (Goksel and Kerslake, 2005). Example 1 is illustrative:

*Example 1:*

**Table:** 

*Çocuk*	*top-un-u*	*al-ıp*	*ev-e*	*git-ti.*
*Child*	*ball-POSS-ACC*	*take-CONV*	*house-DAT*	*go-PAST(3P.SG)*
*Having taken the ball, the child went home.*				

Previous research on the bilingual acquisition of converbial constructions demonstrated that HSs of Turkish diverged from the monolingual baseline in that they tended to use converbs significantly less than the monolingual control group, create sentences where both finite verbs and converbs were used with the subject, which caused ambiguity, as well as place converbs in a detached position loosening the relationship between the converb and the finite verb (Rehbein and Herkenrath, 2015; Turan et al., 2020).

### Evidentiality

Evidentiality is a grammatical category that indicates the source of information (Chafe-Nichols, 1986; Aikhenvald, 2004). In Turkish, tense-aspect-modality markers of *–DI* or *–mIş* are used on predicates as evidentiality markers (Aksu-Koç and Slobin, 1986; Aksu-Koç, 1988). *–DI* is used to indicate that the speaker observed/experienced the event (Example 2), while *–mIş* is used to mark indirect experience for cases when events were not observed by the speaker (Example 3).

*Example 2:*

**Table:** 

*Çocuk*	*pasta-yı*	*ye-di.*
*Child*	*cake-ACC*	*eat-PAST(3P.SG)*
*The child has eaten the cake.*		

*Example 3:*

**Table:** 

*Çocuk*	*pasta-yı*	*ye-miş.*
*Child*	*cake-ACC*	*eat-EVD(3P.SG)*
*The child has eaten the cake.*		

Depending on the context, -*mIş* may be used to indicate that the information is obtained from another person (hearsay/reportative) or inferred by relying on resultative evidence.

A number of studies demonstrated that HSs tended to replace the indirect evidentiality forms with direct ones, ignoring the source of information, and shift between the two even though there were no reasons for that (e.g., Arslan and Bastiaanse, 2014). Furthermore, HSs were reported to be slower and less sensitive to violations than the monolingual baseline. Evidentiality marking also diverged from the baseline in the (re-)activated heritage Turkish of returnees after residing many years in Turkey (Kaya-Soykan et al., 2023).

### Case-marking on direct objects

Case-marking on direct objects is a morphology-syntax-pragmatics interface with two options: (1) the accusative-case ending-I, which, depending upon the preceding vowel sound in the stem and the syllable-final phoneme (i.e., whether it is a vowel or a consonant), may have eight different forms (*İ, I, U and Ü, and (y)İ, (y)I,* and *(y)U* and *(y)Ü*), and (2) the zero-case ending, in which the form of the direct object is identical with the nominative form of nouns. Four contexts determining the case-marking of direct objects have been defined (Enç, 1991; Kornfilt, 1997; Goksel and Kerslake, 2005; Johanson, 2006). A direct object is accusative-marked if it is definite and specific, that is, being a subset of or standing in some recoverable relation to a familiar object (Enç, 1991, p. 24). A direct object is also accusative-marked if it is indefinite/non-specific and appears before the predicate but not in the closest position to it. Finally, a direct object is zero-case marked if it is indefinite/non-specific and appears in the closest position before the predicate in the sentence.

Thus, the speaker marks a direct object depending on its syntactic position and the oppositions [±specific] and [±definite] determined by the discourse and speaker–listener knowledge.

Early and late Turkish L2 users also encounter difficulties in case-marking on direct objects even at advanced levels of proficiency and independently from their L1 backgrounds (Gürel, 2000; Altunkol and Balcı, 2013; Antonova-Unlu and Wei, 2020). Case-marking on direct objects also diverged from the baseline in the (re-)activated heritage Turkish of returnees after residing many years in Turkey.

## Methodology

### Tools

A background questionnaire and two tasks (a c-test and an error correction task) were utilized to measure the attainments of the returnees in their (re-)activated heritage Turkish. The c-test and the error correction task were developed by an expert in testing and validated by two instructors in Turkish, who were also native speakers of the language.

### Background questionnaire

The information about the participants was obtained from their responses to the questionnaire. Self-reports have been often used in bilingual research for assessing the background and linguistic profiles of bi−/multilinguals (Marian et al., 2007; de Bruin et al., 2017; Anderson et al., 2018; Marian and Hayakawa, 2021). The questionnaire consisted of 30 questions regarding the background of the returnees: their gender, place and date of birth, levels of education, family status, the age of moving to and returning from Germany, the quantity of Turkish language use, the number of social contacts while in Germany, and perceived levels of proficiency in both languages at the moment of returning to Turkey and at the present time. A 5-point Likert scale was used to rate the use of the Turkish language as 1—“never,” 2—“seldom,” 3—sometimes,” 4—“often,” and 5—“always.”

### C-test

The c-test has been proven to be a useful and reliable tool for measuring holistic proficiency in foreign and native languages (Klein-Braley, 1985; Dörnyei and Katona, 1992; Chapelle, 1994; Koller and Zahn, 1996; among others) as it requires language users to incorporate knowledge from all linguistic levels. Moreover, c-tests have also been used in previous studies investigating heritage Turkish (re-)activation (Daller and Yıldız, 1995; Treffers-Daller et al., 2016).

The c-test used in this study consisted of two authentic texts chosen from the reading materials of the advanced level (C2) of the Turkish teaching coursebook *Hitit* (Uzun, 2018). Text 1 consisted of 263 words and 17 sentences, and text 2 consisted of 248 words and 15 sentences. The expert deleted 20 items in each of the texts. In all but two cases, the deleted item was every 10th word of the text. The two cases that have been decided as unsuitable for deletion included a proper name and a coordinating conjunction *ve (and)* for simplicity. The participants were requested to fill in the gaps with a suitable word. The missing words implied the use of roots and/or roots and inflectional and derivational morphemes. There were 20 roots and 8 derivational and 26 inflectional morphemes required in the first text, and 20 roots and 9 derivational and 27 inflectional morphemes required in the second text.

The c-test was piloted on 10 native speakers of Turkish. The test–retest reliability coefficient was calculated as 0.91 over a period of 3 weeks. The participants were requested to fill in the gaps with a suitable word. The c-test was scored using the acceptable method (Alderson, 1979) by which gaps were expected to be filled not with the exact word from the original text but with any appropriate word. For example, in the sentence below taken from the c-test, both *kelimelerden (from words)* and *tanımlardan (from definitions)* would be correct. Although the word *tanımlardan* was used in the original text, both variants were accepted as correct when assessing the performance of the participants in the c-test.

*Example 4:*

*Toplum temel birimi olan ailenin yaşadığı ev için Türkçedeki 1. **_______________** biri “huzur ve sükûnet içerisinde yaşanılan yer” anlamında kullanılmakta olan “mesken” dir.*

*One of the Turkish words for the house where the family, the basic unit of society, lives is “residence,” which is used to mean “the place where you live in peace and tranquility.”*

### Error correction task

The error correction task (ECT) has been proven useful for assessing grammar knowledge, especially of specific domains/structures (Azar, 2007). The ECT was used in this study to examine the perception of grammatical and ungrammatical uses of Turkish by the returnees and their ability to produce the correct forms. The task consisted of 30 ungrammatical and 30 grammatical items. The items covered three morphosyntax domains (two of which also required the activation of pragmatics) that have been reported as vulnerable in the available research on heritage Turkish acquisition and (re-)activation: evidentiality, direct object case-marking, and converbial constructions (Arslan and Bastiaanse, 2014; Akkus et al., 2017; Turan et al., 2020; Kaya-Soykan et al., 2023).

The participants were requested to judge the task items regarding their grammaticality, as grammatically correct or incorrect, and correct them if considered incorrect. The task was piloted on 10 native speakers of Turkish. The test–retest reliability coefficient was 0.92 over a period of 3 weeks.

### Participants

The study participants were 28 Turkish-German bilinguals (*Women* = 18 and *Men* = 10) whose ages varied from 19 to 59 years (*M* = 32.79, *SD* = 11.24). As for the educational level of the participants, 9 were university students, 11 were university graduates, 7 had a PhD degree, and 1 had an MA degree. Among all the participants, 9 were studying German language and literature or German translation and interpreting in Turkey, 9 were instructors of German at universities, 3 participants were employees of a firm, 2 were working as German language specialists at ministries, and 5 were unemployed at the moment of the data collection.

A total of 22 participants were born in Germany and 6 were born in Turkey. Among those 6 participants who were born in Turkey, the age during the move to Germany ranged from 3 to 7 years. Thus, the average age of the returnee participants during the move to Germany was approximately 1 year (*M* = 1.04, *SD* = 2.16).

Both parents of all the participants were Turkish and had lived in the Central Anatolian region before moving to Germany. The communication among the family members was in Turkish. The onset of participants’ formal contact with the German language varied from the age of 3 years, when they started a German kindergarten, to the age of 6 years, when they started a primary school in Germany (*M* = 4.46, *SD* = 1.78). As for secondary school education, 12 participants reported that they completed their secondary school education in Germany, 9 studied the final 2 years of secondary school in Turkey, and 7 participants finished their secondary school in Turkey. As for high school education, 24 participants received it in Turkey, 3 in Germany, and 1 started a high school in Germany but finished in Turkey. All the participants pursued their university education at various departments in Turkey.

All the participants stated that they had used Turkish while they were in Germany to varying degrees from “seldom” to “always.” The participants also indicated the social groups (parents, relatives, neighbors, friends, and teachers) with whom they had been using Turkish while in Germany. The sum of the latter two variables (the frequency of Turkish use, from 1 for “seldom” to 5 for “always,” and the number of interlocutors) was defined as the perceived frequency of Turkish language use in the German-dominant context.

The participants’ age when returning to Turkey varied from 7 to 20 years (*M* = 13.64, *SD* = 3.18). The length of residence of the returnee participants in Germany varied from 6 to 20 years (*M* = 12.68, *SD* = 3.83). At the time of the study, the participants had been residing in Turkey for 5–45 years (*M* = 18.96, *SD* = 12.05). All the participants reported that after returning to Turkey, they had been using Turkish daily in all public places and interacting with family members, friends, and colleagues. All the participants reported that their Turkish had improved significantly after return, and they all considered themselves as monolingual-like in Turkish in the four skills: speaking, listening, writing, and reading.

### Control group

The control group consisted of 28 Turkish speakers (*Women* = 22 and *Men* = 6) who had lived all their lives in Turkey and whose ages varied from 18 to 60 years (*M* = 30.17, *SD* = 10.56). As for the educational level of the participants, 3 were university students, 9 were university graduates, 9 had an MA degree, and 7 had a PhD degree. All the participants in the control group were from the Central Anatolian region.

## Data analysis and results

### C-test

Descriptive statistics were used to examine the performance of the returnee participants and the control group on the c-test (see Table 1).

**Table 1:** Performance of the returnee and control groups on the c-test.

	Returnee group	Control group
Minimum	25.00	38.00
Maximum	40.00	40.00
Mean	36.21	39.68
SD	3.83	0.61
Median	36.5	40.00

The data analysis showed that the mean score of the returnee participants on the c-test was 36.21 (91%). The results of the returnee group (*M* = 36.21, *SD* = 3.83) were significantly different (*W* = 134, *p* < 0.000) from the results of the control group (*M* = 39.68, *SD* = 0.61) when compared with the help of the Wilcoxon signed-rank test (RStudio Team, 2020).

### Error correction task

Descriptive statistics were used to examine the performance of the returnee participants and the control group on the ECT (see Table 2).

**Table 2:** Performance of the returnee and control groups on the error correction task.

	Returnee group	Control group
Minimum	21.00	26.00
Maximum	30.00	30.00
Mean	26.14	28.8
SD	2.95	1.21
Median	26.00	29.00

The data analysis showed that the mean score of the returnee participants on the error correction task was 26.14 (87%). The results of the returnee group (*M* = 26.14, *SD* = 2.95) were significantly different (*W* = 181.5, *p* = 0.000) from the results of the control group (*M* = 28.8, *SD* = 1.21) when compared with the help of the Wilcoxon signed-rank test (RStudio Team, 2020).

Furthermore, the performance of the returnee participants was examined for each of the three domains included in the error correction task separately. Table 3 presents the descriptive statistics for each of the domains.

**Table 3:** Performance of the returnee group on the domains.

	Evidentiality	Direct object marking	Converbs
Minimum	5.00	4.00	9.00
Maximum	10.00	10.00	10.00
Mean	8.071	8.071	9.964
SD	1.804	1.585	0.1890
Median	8.00	8.00	10.00

**Figure 1:**
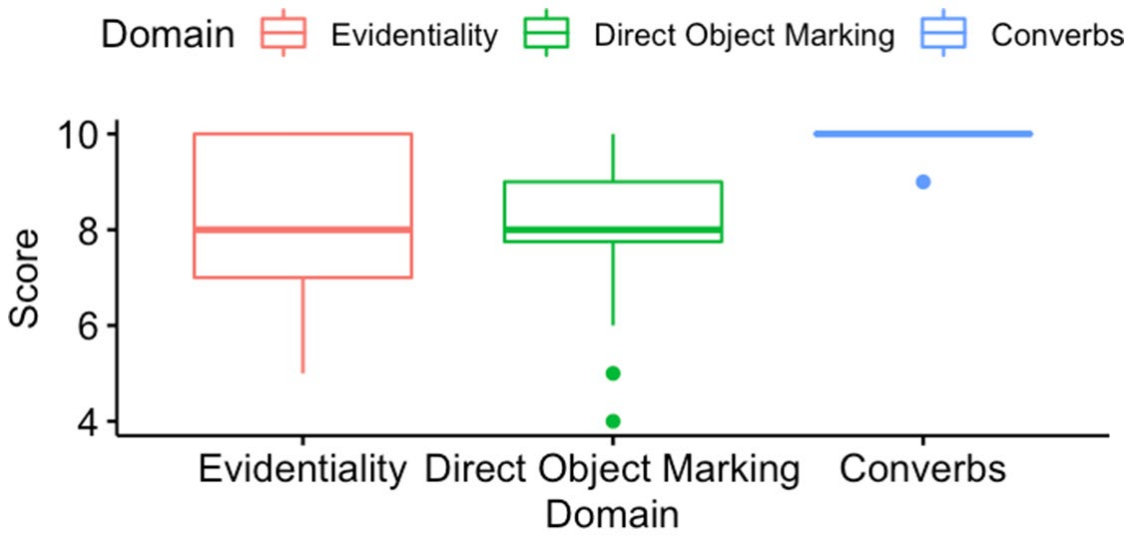
Performance of the returnee group on the domains.

The Kruskal–Wallis rank sum test was run to see whether there was a significant difference in the performance of the returnee participants in the domains of evidentiality, direct object marking, and converbs. The Kruskal–Wallis rank sum test showed that the returnee participants performed significantly differently on the three domains (*H* (2) = 32.922, *p* < 0.000). Pairwise comparisons using the Wilcoxon rank sum test with continuity correction were used to compare the returnee participants’ performance in all the three domains. The difference between the domain of converbs and the two other domains of evidentiality and direct object markings was significant (*p* < 0.000), while the performance of the returnee participants on the domain of evidentiality did not differ (*p* = 0.9) from their performance on the domain of direct object marking as shown in Figure 1.

When the performance of the returnee group was compared with the results of the control group for each of the domains using the Wilcoxon rank sum test, significant differences were revealed between the returnee group and the control group in the domains of evidentiality (*W* = 226, *p* = 0.004) and direct object marking (*W* = 224.5, *p* = 0.004), while no difference was found between the groups in the converb domain (*W* = 406, *p* = 0.571).

The data analysis also showed that there were five returnee participants whose scores on the tasks were compatible with the mean of the control group. Two of these participants moved to Germany at the ages of 6 and 7 years and returned to Turkey when they were 12 and 13 years old; the other two participants were born in Germany and returned to Turkey when they were 6 and 7 years old, and the last participant moved to Germany at the age of 1 year and returned to Turkey at the age 7 years. Thus, the average length of these participants’ residence in Germany was approximately 6 years (*M* = 6.6, *SD* = 0.548).

In addition to these five participants, there were six other returnee participants who scored within the minimum–maximum range of the control group (*min* 65 out of 70 for both tasks). The age at the return of these six participants varied from 11 to 20 years (*M* = 14.17; *SD* = 3.31); however, all six participants reported higher than the group average values for the independent variable of frequency of Turkish language use in the migration context, which varied from 8 to 10 years (*M* = 9.00, *SD* = 0.89).

Along with the overall divergence of the returnee group from the baseline, 39% of the returnees performed compatibly with the control group. This finding, together with a pretty high standard deviation for both tasks, suggests the impact of external factors on the attainments in the reactivated HL.

## Effect of sociolinguistic/external factors on the returnees’ attainments in the reactivated heritage Turkish

As previously mentioned, one of the primary objectives of this study is to explore the connection between the linguistic outcomes of returnees and their linguistic experiential factors. To achieve this, we employed the random forest method (Breiman, 2001) and implemented it using the *Ranger* package (Wright and Ziegler, 2017). This analysis aimed to assess how sociolinguistic factors influence the cloze test and error correction scores of the returnees.

Random forests are built upon decision trees, which employ a series of binary rules to predict a response variable. Decision trees, used with numerical and categorical response variables, are statistical models that employ recursive partitioning as their primary algorithm. Put more simply, the algorithm initially tests the association of independent variables with the response variable. If it identifies multiple independent variables associated with the response variable, the model assesses the strength of each association. The variable with the strongest association is selected for the initial binary split. For instance, if the independent variable is binary with values “M” and “F,” one subset will comprise all observations with the “M” value, while the other subset will include those with the “F” value. Each subset forms a branch in the tree. This process is iteratively repeated until all independent variables have been evaluated. A random forest is constructed by aggregating a large number of decision trees. To create diverse trees, random forests employ two key procedures: bootstrap aggregating and random predictor subset selection.

Bootstrapping involves generating subsamples of the dataset with replacement, allowing each observation to be chosen more than once in a subsample. Consequently, the subsample contains two-thirds of the observations, while the remaining one-third constitutes the out-of-bag sample. Each tree in a random forest is trained on a distinct bootstrapped sample.

Random predictor selection refers to the procedure in which the algorithm chooses a random subset of predictor variables to train each tree in the forest, denoted as “mtry.” For categorical predictors, this value is typically the square root of the total number of predictor variables, whereas for continuous predictors, it is the number of predictors divided by 3 (Hastie et al., 2001; Strobl et al., 2009).

The choice of random forest over more traditional analyses, such as linear regression, was based on two main reasons: the high number of predictor variables derived from the questionnaire, which is more than the number of participants, meaning that there are more predictors than observations, a problem usually known as *p > n*. Linear regression models are not recommended in this scenario (Bühlmann and van de Geer, 2011; Chakraborty et al., 2012). The second reason is the fact that several of the questions were highly correlated. The presence of correlated variables would have made the results uninterpretable and inaccurate. Since we were interested in determining the effect of each of the variables targeted in the questionnaire, we did not want to do a principal component analysis, because this type of analysis, while taking care of the correlation among the variables, obscures the effect of the individual predictors.

Random forests can handle scenarios with more predictors than observations and manage correlated predictors. They are versatile, accommodating both continuous and categorical predictors, and are robust to variable scaling. Additionally, random forests provide variable importance rankings, helping identify the most significant predictors. The Ranger package’s random forest implementation adds the ability to calculate value of ps, enhancing our ability to assess the statistical significance of each variable’s contribution to explaining the outcome.

The Ranger package offers two value of p calculation methods. We opted for the Altmann method (Altmann et al., 2010), which involves performing 1,000 permutations, recommended for greater precision by the Ranger package creators.

We ran two random forest analyses, one for each of the two scores: cloze test and error correction. Each random forest consisted of 5,000 trees and employed the default *mtry* value, which is the square root of the number of predictors. In total, each random forest included 23 variables after removing the surplus variables. A complete list of the variables is available online.

## Results of random forest models

### Cloze test

The next model in Table 4 shows the results of the cloze test. The model determined that *frequency of Turkish use* (*p* < 0.05) is the most important predictor of cloze test performance, followed by *Turkish use* (*p* < 0.05), *years spent in Turkey* (*p* < 0.05)*, years spent in Germany* (*p* < 0.05), *and Turkish use* (*p* < 0.05).

**Table 4:** Significant predictor variables for the cloze test.

Variable	Importance	Importance #	*p*-value	*p* significance
Fr_of_Tur_Use	1.441432	1	0.01998	*p* < 0.05
Tur_use	1.339889	2	0.023976	*p* < 0.05
Years_in_Tur	1.313394	3	0.03996	*p* < 0.05
Years_in_Ger	1.279082	4	0.028971	*p* < 0.05
Tur_class	1.008857	5	0.020979	*p* < 0.05

**Figure 2:**
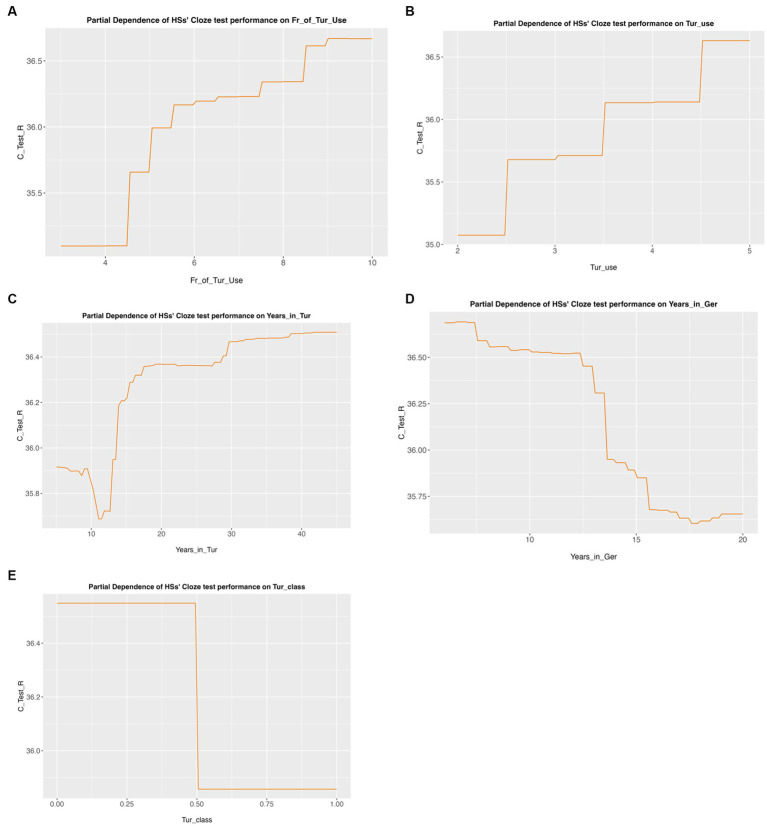
Partial dependence plots of returnees’ cloze test performance. **(A)** Partial dependency of cloze test performance on the frequency of Turkish use. **(B)** Partial dependency of cloze test performance on Turkish use. **(C)** Partial dependency of cloze test performance on years spent in Turkey. **(D)** Partial dependency of cloze test performance on years spent in Germany. **(E)** Partial dependency of cloze test performance on Turkish classes.

In Figure 2, we show partial dependence plots of returnees’ cloze test performance on the five variables selected as significant by the model. Figure 2A shows that as returnees’ frequency of use of Turkish increases, so does their task. Similarly, in Figure 2B, it is shown that a higher use of Turkish results in a higher task score. Figure 2C shows that if the returnees have spent more time in Turkey, then their task performance increases too. Figure 2D shows that spending more time in Germany will have a negative effect on returnees’ performance. And finally, in Figure 2E, we see that those returnees who claimed that they did not have any Turkish classes outperform those who did.

### Error correction

In Table 5, we show the random forest for the error correction task. The most important predictor, in this case, is the time a returnee spent in Germany (*p* < 0.05), followed by whether they had any Turkish classes (*p* < 0.05). The final most important variable is the amount of Turkish use (*p* < 0.05).

**Table 5:** Significant predictor variables for the error correction task.

Variable	Importance	Importance #	*p*-value	*p* significance
Years_in_Ger	1.60235613	1	0.002997003	*p* < 0.05
Tur_class	0.72749702	2	0.017982018	*p* < 0.05
Tur_use	0.4220541	3	0.047952048	*p* < 0.05

**Figure 3:**
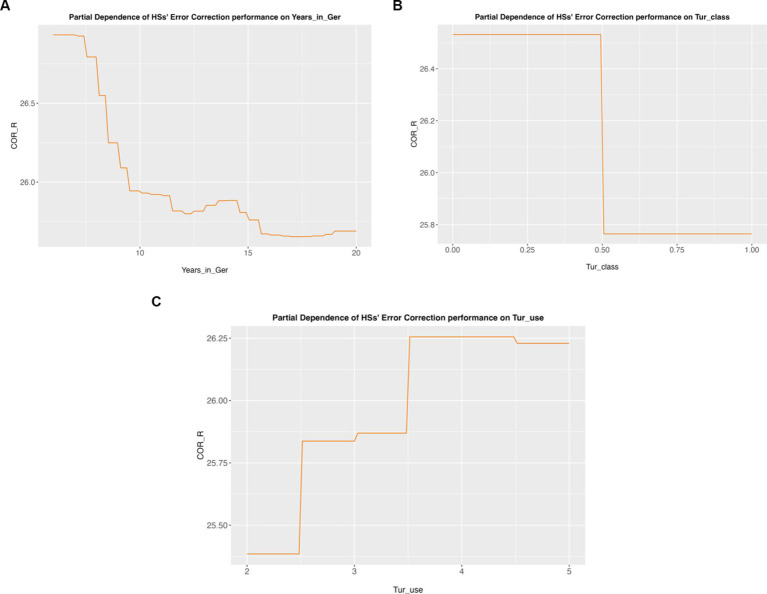
Partial dependency plots for the error correction task. **(A)** Partial dependency of error correction performance on years spent in Germany. **(B)** Partial dependency of error correction performance on Turkish class attendance. **(C)** Partial dependency of error correction performance on the use of Turkish.

As above, Figure 3 shows the partial dependence plots for the error correction task. We observe in Figure 3A that there seems to be a negative relationship between the time spent in Germany and task performance. That is, the more time a returnee spends in Germany, the lower their error correction task performance. Similarly, in Figure 3B, we see that attending Turkish classes also has a negative effect on the returnees’ performance in this task. Figure 3C shows that the more Turkish returnees use Turkish, the more likely it is that they perform better in the error correction task.

## Discussion

Most of the evidence regarding individual variation in the heritage speaker bilingualism research comes from research on *typical* heritage speakers: those who are born and/or grow up in a dominant host language environment with their home language(s) differing from the societal one (Rothman, 2009; Kupisch and Rothman, 2018; Polinsky and Scontras, 2019). Examining the reactivation of returnees’ heritage language upon re-exposure to the home environment offers valuable contributions to our understanding of heritage language development and maintenance, and reactivation, as well as the influence of various factors on the attainment of language competence among returnee heritage speakers.

### Selective vulnerability of grammatical domains

The analysis of the cloze test and error correction task revealed significant differences in performance between the returnees and the control group. These findings highlight the selective vulnerability of different grammatical domains when heritage speakers return to their country of origin. The cloze test and error correction task demonstrated that certain linguistic domains, especially when more than one domain of grammar is involved, such as interfaces of syntax and pragmatics (evidentiality and direct object marking), can be more susceptible to change, as evidenced by the significant differences between the returnee group and the control group in these domains. In contrast, other domains of the grammar morphosyntax domain, represented by converbs in this particular study, appeared to exhibit greater resilience, with no significant difference observed between the two groups. These results are in line with previous research (e.g., Treffers-Daller et al., 2007; Kaya-Soykan et al., 2023) that showed that not all linguistic structures may be (re-)activated and converge toward the baseline for granted once a HS is immersed in the environment where the HL is dominant again.

From a cross-linguistic influence perspective, the category of evidentiality is not available in German, the source of information is marked lexically (Diewald and Smirnova, 2010; Haßler, 2015), and the category of definiteness and specificity, which is involved in direct object marking in Turkish, is similarly available in German grammar (Dodd et al., 2003). Nevertheless, no benefit in the performance of the returnee participants in the domain of direct object marking has been revealed in comparison with the domain of evidentiality. Such language behavior of the returnees in their (re-)activated HL might be considered as a piece of evidence supporting the view that restricted resources of bilinguals in integrating information from different modules but not (only) cross-linguistic influence is the underlying reason for the vulnerability in different domains of grammar (Hopp, 2009; Antonova-Unlu and Wei, 2020).

### Experiential factors and individual variability

Our findings offer valuable insights into the role of specific experiential factors in explaining individual variability observed with the returnee group. Our study aligns with recent trends in bilingualism (e.g., DeLuca et al., 2019; Rothman et al., 2023) as well as heritage speaker bilingualism research (e.g., Rodina et al., 2020; Bayram et al., 2021; Tomić et al., 2023), recognizing the dynamic and complex nature of bilingual experiences and understanding heritage speakers within their own right (Polinsky and Scontras, 2019).

### Role of formal language education

In this line, the findings shed light on the potential influence of formal language education in the heritage language. Returnee heritage speakers who reported not attending Turkish classes outperformed those who did in the cloze test and the error correction task. While formal language education has the potential to impact heritage language performance positively (e.g., Kupisch and Rothman, 2018; Bayram et al., 2019, 2021; Gharibi et al., 2023), the results of this study are nuanced. The negative effect observed in both tasks suggests that formal education may not always align with enhanced performance, reinforcing the necessity to develop comprehensive heritage language maintenance strategies that go beyond formal education. While formal language education can be beneficial in certain contexts, it may not always guarantee enhanced performance, suggesting that the effectiveness of these programs may vary depending on individual circumstances.

### Frequency of language use and sociolinguistic factors

These findings have significant implications for heritage language maintenance and (re-)activation. The research underscores the importance of consistent language use and maintaining sociolinguistic networks. The frequency of Turkish language use emerged as a critical factor influencing the (re-)activation and proficiency in the heritage language after returning to the home language environment. Returnee participants who reported using Turkish more frequently and having a broader social network for communication in Turkish exhibited advantages in heritage Turkish (re-)activation after returning to their home country. This finding highlights the significance of creating opportunities for heritage speakers to engage in regular language use, even when they are outside the heritage language-dominant environment.

### Residence in non-heritage language-dominant environments

The findings also reveal the potential challenges faced by heritage speakers who spend prolonged periods in non-heritage language-dominant environments. The length of time spent in a non-heritage language environment, as revealed by the analysis, negatively correlates with task performance (see Figure 2A). A longer residence in the context where another language is dominant implies fewer opportunities to get sufficient input into and use of the HL. This suggests that heritage speakers may experience difficulties in maintaining their heritage language competence when exposed to prolonged periods in a non-heritage language-dominant environment. These findings are consistent with numerous previous studies (Hoff and Naigles, 2002; Gutiérrez-Clelle and Kreiter, 2003; Gathercole and Thomas, 2005; Blom, 2010; Rodina and Westergaard, 2015; Unsworth, 2015; Correia and Flores, 2017 among others) demonstrating that the amount of input is a significant predictor of language development, especially in contexts where the language is not supported by the community. It is possible to suggest that the returnee participants who reported that they had used Turkish more frequently and had a wider social network to communicate in Turkish acquired their heritage Turkish in Germany better and, by extension, had an advantage in the HL (re-)activation after their return to the country of origin.

## Conclusion

In summary, the findings from this study offer important implications for heritage language maintenance and (re-)activation. The results underscore the dynamic and multifaceted nature of heritage language development, emphasizing the influence of both internal linguistic structures and external factors such as language use frequency and formal education. These findings offer valuable insights for developing strategies to support heritage speakers in preserving, activating, and enhancing their heritage language proficiency.

Ultimately, this study contributes to the broader discourse on heritage language development and acknowledges the unique linguistic journeys that heritage speakers undertake. The research encourages a more comprehensive and individualized approach to heritage language (re-)activation, recognizing that heritage speakers are not simply recipients of their linguistic environment but active agents in shaping their bilingualism.

## Data availability statement

The raw data supporting the conclusions of this article will be made available by the authors, without undue reservation.

## Author contributions

EA-U: data collection, handling and analysis, conceptualisation, and writing. FB: data analysis, conceptualisation, and writing. All authors contributed to the article and approved the submitted version.
